# Technical and Economic Comparison between Sodium and Ammonium Agents in the Jarosite Precipitation Process—An Evaluation for Industrial Applications

**DOI:** 10.1021/acsomega.3c03536

**Published:** 2023-09-12

**Authors:** Ali Asimi Neisiani, Farhad Moosakazemi, Saeed Chehreh Chelgani

**Affiliations:** † Department of Mining and Metallurgical Engineering Yazd University, Yazd 8915818411, Iran; ‡ Bafgh Zinc Smelting Company (BZSC), Yazd 8915818411, Iran; § Chemical Engineering Department, Laval University, Québec G1 V 0A6, Canada; ∥ Minerals and Metallurgical Engineering, Department of Civil, Environmental and Natural Resources Engineering, Swedish School of Mines, Luleå University of Technology, Luleå SE-971 87, Sweden

## Abstract

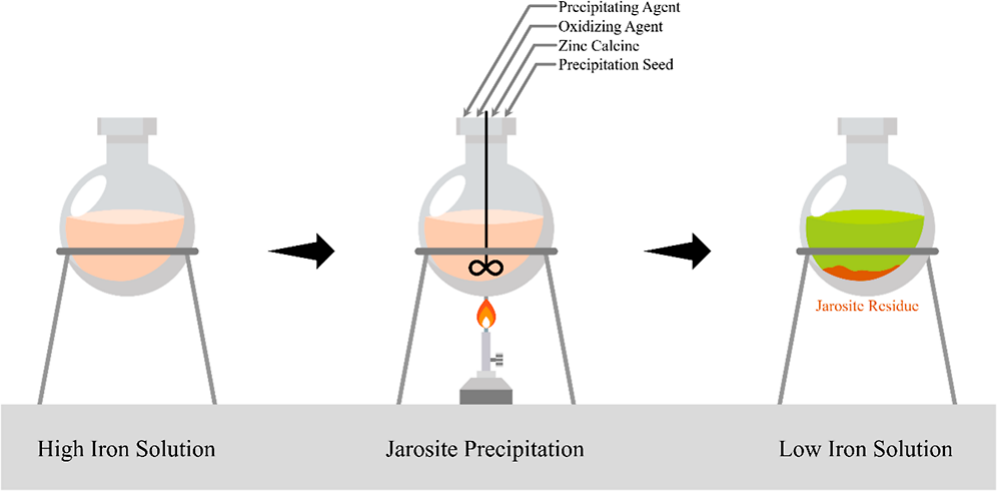

Iron content can cause severe challenges through zinc production from zinc sulfide concentrate. The zinc industry extensively uses the jarosite precipitation process (JPP) to precipitate dissolved iron and remove it before transferring the solution to downstream stages. Precipitating agents (PAs) play an essential role in the JPP. However, surprisingly, no study compares the efficiency of various PAs on an industrial scale. As an innovative approach, this investigation compares the technical and economic aspects of using various sodium and ammonium compounds (hydroxides, carbonates, bicarbonates, sulfates, and bisulfates) as typical PAs for the JPP at the Bafgh Zinc Smelting Company (BZSC) plant. Experimental results revealed that ammonium hydroxide, with 90.85% iron removal efficiency, had the highest performance, and sodium bisulfate and ammonium bisulfate had the lowest efficiency (74.54 and 77.13%, respectively). However, since ammonium hydroxide is a corrosive PA, it is not a promising alternative to sodium sulfate (with both economic and safety issues). Based on technical and economic assessments, sodium carbonate (84.31% iron removal efficiency) showed the highest potential for an efficient JPP.

## 1 Introduction

During zinc production, since zinc sulfide concentrates [for example, sphalerite: (Zn, Fe)S] usually have a strong metal and mineral association with iron as pyrite (FeS_2_) or pyrrhotite [Fe_(1–*x*)_S, *x* = 0–0.2], iron removal plays an important role.^1–3^ At present, more than 80% of the world’s zinc production is obtained by hydrometallurgical processes.^4–6^ Roasting-Leaching-Electrowinning (RLE) is the most known hydrometallurgical process. During the RLE process, when zinc sulfide concentrate is roasted, zinc ferrite (ZnO·Fe_2_O_3_) is produced as an unavoidable compound (eqs 1 and 2).^1,7,8^ Thus, zinc ferrite formation is directly proportional to the iron content in the zinc concentrate. Zinc ferrite is barely dissolved in the neutral leaching step, resulting in extensive amounts of neutral leaching residues. Acid leaching is required to dissolve zinc ferrite to enhance the process’s efficiency. However, the concentration of iron in the produced zinc sulfate solution (ZSS) is always extremely high due to the destruction of the zinc ferrite, stable structure, and simultaneous iron dissolution (eq 3).^1,9,10^
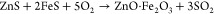
1
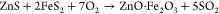
2
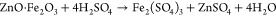
3

For Fe removal from the ZSS, prior to transferring the solution to downstream stages, including purification and electrowinning, ferrous ions (Fe^2+^) present in the ZSS must initially be converted to ferric ions (Fe^3+^) by an oxidizing agent, precipitated, and removed from the solution as an iron compound such as jarosite, goethite, or hematite. The most widely used oxidizing agent in this part of the zinc industry is manganese dioxide, which can convert Fe^2+^ to Fe^3+^ based on eq 4. Therefore, purification procedures such as the jarosite precipitation process (JPP), cementation, and solvent extraction are the most potent methods to control the concentration of impurities.^9,10^
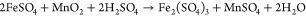
4

JPP is the most widely utilized technique in hydrometallurgy for Fe rejection from leaching solutions. JPP has significant merits, such as cost-effectiveness, easy operation, and readily filterability of precipitated residue.^11–14^ At zinc smelters that use JPP, the purpose of the process is not to eliminate the whole iron content of the ZSS since it is required to remain part of the dissolved iron in the solution to be transferred to the neutral leaching step (to remove some impurities through coprecipitation with iron hydroxide and diminish their concentrations to acceptable ranges).^1,11^ During JPP, ferric ions are chemically precipitated from ZSS (eq 5) as a compound, which is illustrated as AFe_3_(SO_4_)_2_(OH)_6_, where A can be various monovalent cations such as Na^+^, NH_4_^+^, K^+^, Rb^+^, Ag^+^, and Ti^+^.^15–18^ Therefore, different precipitating agents (PAs) were added to the process to enhance the JPP and iron removal efficiency (Table 1). It has been reported that the type of monovalent cations plays a major role in the JPP’s performance and can influence the kinetics of the jarosite precipitation reaction (K^+^ > NH_4_^+^ > Na^+^ > H_3_O^+^). Meanwhile, sodium and ammonium compounds are more affordable and available than potassium compounds and consequently more extensively utilized in the zinc hydrometallurgy industry.^19,20^
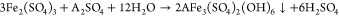
5

**Table 1:** Various PAs and Their Characteristics

type	compound	molar mass	jarosite precipitation reaction
hydroxide	(NH_4_)OH	35.05	2AOH + 3Fe_2_(SO_4_)_3_ + 10H_2_O → 5H_2_SO_4_ + 2AFe_3_(SO_4_)_2_(OH)_6_↓
	NaOH	40.00	
carbonate	(NH_4_)_2_CO_3_	96.09	A_2_CO_3_ + 3Fe_2_(SO_4_)_3_ + 11H_2_O → 5H_2_SO_4_ + CO_2_ + 2AFe_3_(SO_4_)_2_(OH)_6_↓
	Na_2_CO_3_	105.99	
bicarbonate	(NH_4_)HCO_3_	79.06	2AHCO_3_ + 3Fe_2_(SO_4_)_3_ + 10H_2_O → 5H_2_SO_4_ + 2CO_2_ + 2AFe_3_(SO_4_)_2_(OH)_6_↓
	NaHCO_3_	84.01	
sulfate	(NH_4_)_2_SO_4_	132.14	A_2_SO_4_ + 3Fe_2_(SO_4_)_3_ + 12H_2_O → 6H_2_SO_4_ + 2AFe_3_(SO_4_)_2_(OH)_6_↓
	Na_2_SO_4_	142.04	
bisulfate	(NH_4_)HSO_4_	115.11	2AHSO_4_ + 3Fe_2_(SO_4_)_3_ + 12H_2_O → 7H_2_SO_4_ + 2AFe_3_(SO_4_)_2_(OH)_6_↓
	NaHSO_4_	120.06	

Several investigations have been carried out to understand the performance of different PAs and additives used in JPP, such as precipitation seeds,^14,21,22^ neutralizing,^11,23–26^ and oxidizing agents.^27–29^ However, surprisingly, no investigation has explored or compared the performance of various compounds (PAs), especially on an industrial scale. Therefore, as a unique approach, this study will compare the technical and economic aspects of using various sodium and ammonium compounds for the JPP in the BZSC.

## 2 Materials and Methods

### 2.1 Materials and Reagents

**Figure 1:**
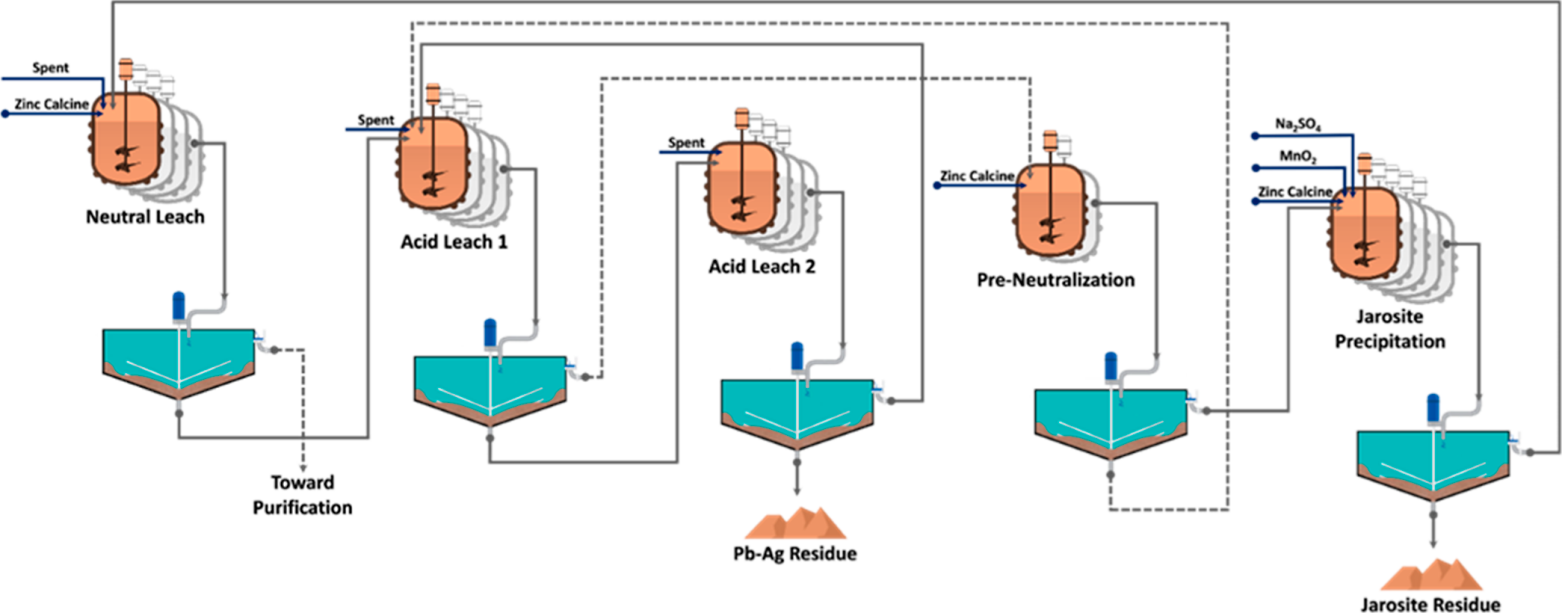
Schematic flowsheet of BZSC’s leaching unit and jarosite precipitation line.

This study utilized the chemical grade additives, including oxidizing agents and precipitating compounds, for all experiments. Zinc calcine (ZC), the product of oxidative roasting of zinc sulfide concentrates used to adjust the pH, and sulfuric acid were provided from the roasting and sulfuric acid production units of the BZSC (Figure 1), respectively. Since H_2_SO_4_ is produced during JPP (eq 5), the pH was adjusted at the beginning of experiments and modified to the incipient value during the reactions at 15 min intervals.

The ZSS samples containing a high amount of iron were provided from BZSC’s jarosite line (inlet solution) for the jarosite precipitation experiments (Table 2). ZC was analyzed by an atomic absorption spectrometer (Varian SpectrAA 220) and X-ray fluorescence spectroscopy (XRF, Rigaku, ZSX Primus II) (Table 3). Besides, the jarosite residue was utilized as precipitation seed. After washing, drying, grinding, and sieving, −200 mesh (−74 μm) jarosite residue was collected from the plant for experimental assessments. In all experiments, MnO_2_ was used to convert all ferrous ions in the solution to ferric ions. In other words, all the iron content in the solution was subjected to a jarosite precipitation reaction.

**Table 2:** Chemical Characterization of the ZSS

concentration of components (g/lit)					
Zn	Fe (total)	Fe^2+^	K	Na	H_2_SO_4_
101.37	9173 × 10^–^^3^	94 × 10^–^^3^	173 × 10^–^^3^	396 × 10^–^^3^	24.75

**Table 3:** Chemical Characterization of the ZC

content of elements (wt %)						
Zn	Fe	Pb	Ca	K	Na	Ag
51.3	3.70	3.21	1.89	0.22	0.26	0.01

### 2.2 Experimental Procedures

The experiment conditions were determined based on the previous investigation^11^ and according to the limitations and conditions prevailing in the BZSC’s production line. Prior to jarosite precipitation tests, solutions’ temperatures increased using a hot plate to approximately 90 °C (optimized value^11^). Since the JPP is sensitive to temperature, a heat-up process was immediately conducted to minimize the jarosite formation in the solution preparation step. Then, the prepared solution was immediately transferred to a 2 L baffled test reactor located in an oil bath to accurately control the temperature (90 ± 1 °C). The glass reactor with three openings for the thermometer, agitator, and sampler was utilized. After reactor contents reached the desired reaction temperature, the pH was adjusted, chemical compounds, including PA, oxidizing agent, and precipitation seed, were added (zero time), and retention time was calculated. The solution samples were agitated by a mechanical stirrer with two 45° pitched blades (blade diameter = 5.5 cm). At determined intervals, samples were taken and immediately brought to ambient temperature to restrict further reactions. The sampling process was performed using a syringe fitted with an in-line filter to avoid collecting any precipitated jarosite particles. Withdrawn samples were quickly transferred from the syringe into a stoppered test tube to diminish evaporation. After cooling, a 5 mL pipet was used to take a precise sample, and then it was analyzed. At the end of the reaction period, the slurries and any material adhering to the vessel were filtered on a Buchner vacuum filter using Albet DP 5893-150 ashless filter paper. Subsequently, a large amount of warm distilled water was used to wash the precipitated jarosite, and then the precipitates were exposed to a temperature of 110 °C for 24 h in an oven to dry completely. Dried jarosite residues were analyzed by using X-ray diffraction (XRD) (D8ADVANCE, Bruker Company, Germany). The scanning range was 10–90°, and the scanning speed was 0.2 (deg)/s. Scanning electron microscopy (SEM) (FESEM-SIGMA VP; ZEISS Company, Germany) was utilized to observe the morphology of the precipitated jarosite at an accelerating voltage of 10 kV.

Based on the plant procedure, the solution’s zinc and acid concentrations were measured by titration with EDTA and NaOH, respectively. The amount of iron was measured using stannous chloride (SnCl_2_) reduction followed by potassium dichromate titration with a sodium diphenyl sulfonic acid indicator (when [Fe^3+^] > 0.1 g/Lit) or by the Varian SpectrAA 220 atomic absorption spectrometer (when [Fe^3+^] < 0.1 g/Lit). At zinc smelters’ plants, including BZSC, the ZC produced in the roasting unit is utilized as an available, suitable, and affordable neutralizing agent.^11,30,31^ JPP was performed by using various PAs to study and compare their performance under listed conditions (Table 4). It should be noted that in all experiments, ZC was used to adjust the pH periodically. The efficiency of iron removal (η_*p*_, percent), the cost of PA (*C*_p_, dollar), and zinc loss (*C*_l_, dollar) were respectively calculated according to
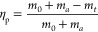
6
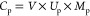
7
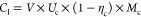
8where: *m*_0_: initial Fe concentration in the inlet solution of the jarosite line (mg/Lit), *m*_a_: Fe concentration added to the solution by neutralizing agent (ZC) (mg/Lit), *m*_t_: Fe concentration in solution at time *t* (mg/Lit), *V*: volume of pulp input to the jarosite line (M^3^/year), *U*_p_: PA consumption (Ton/M^3^), *M*_p_: PA price (dollar/Ton), *U*_c_: ZC consumption (Ton/M^3^), η_c_: The leaching efficiency of ZC in jarosite line, *M*_c_: ZC price (dollar/Ton)

**Table 4:** Conditions for the Jarosite Precipitation Experiments

parameters					
pH	retention time (min)	stirring speed (rpm)	temperature (°C)	jarosite seed (g/lit)	precipitating ion (A^+^) (mol/lit)
1	300	600	90	50	0.03

## 3 Results and Discussion

### 3.1 PAs’ Performance

**Figure 2:**
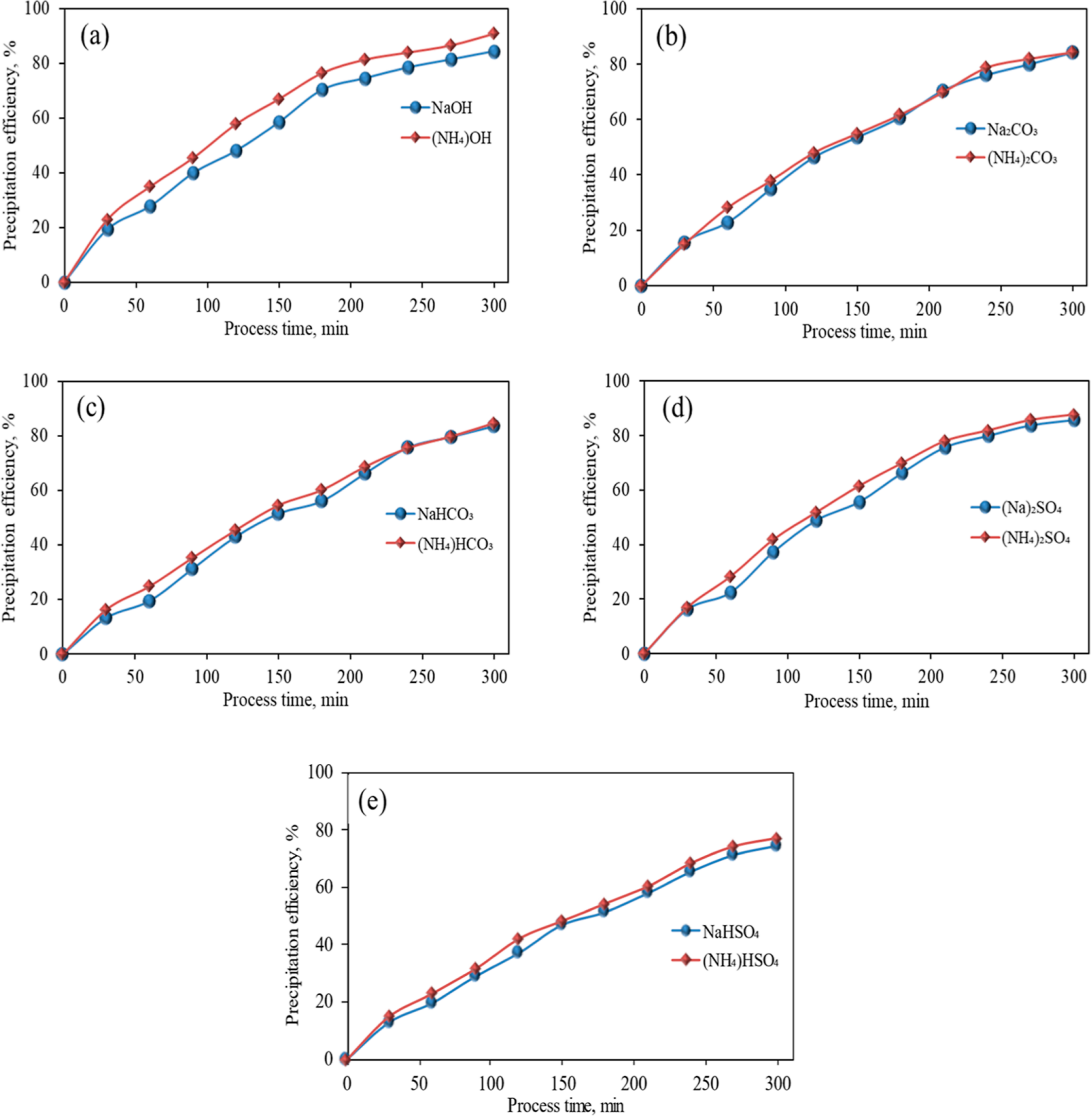
Iron precipitation efficiency of the sodium and ammonium salts of (a) hydroxides, (b) carbonates, (c) bicarbonates, (d) sulfates, and (e) bisulfates in the JPP.

Exploring the performance of various PAs (Figure 2) indicated that under similar conditions, the performance of ammonium sulfate was higher than that of sodium sulfate in removing iron during JPP. This is in accordance with the obtained results in a study conducted by Dutrizac (2010). He has reported that, in the temperature range of 70 to 100 °C, jarosite precipitation improved when (NH_4_)_2_SO_4_ was used instead of Na_2_SO_4_. This improvement was approximately 9 and 18% without and with (25 g/Lit) jarosite seed, respectively.^32^

**Figure 3:**
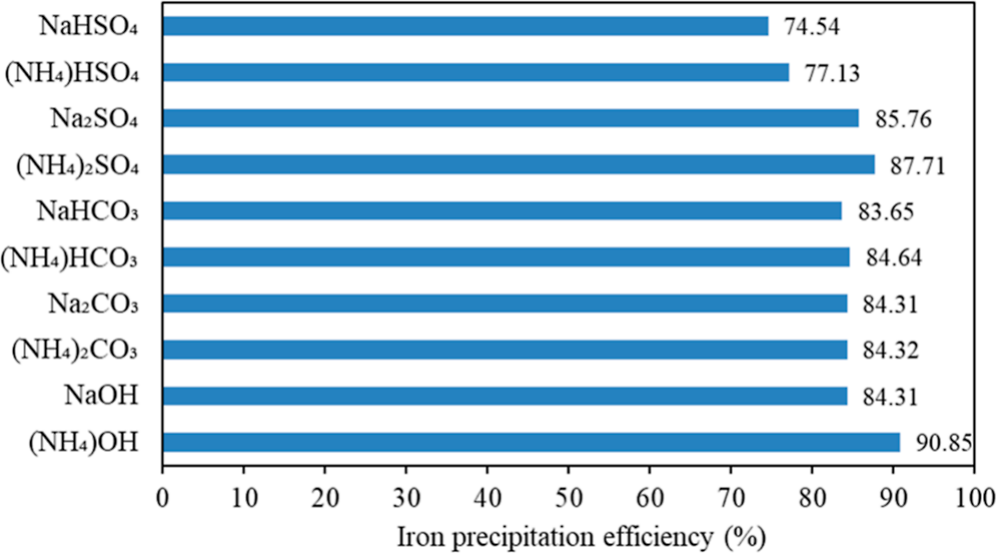
Comparative results of PA’s performance in iron removal at the end of reactions.

In general, the performance of other ammonium compounds, including hydroxide, carbonate, bicarbonate, and bisulfate, was higher than that of sodium compounds at the end of the process under the same conditions (Figure 3). The experimental outcomes exhibited that among all the compounds used as a PA, ammonium hydroxide had the best performance with an iron removal efficiency of 90.85%. On the other side, sodium bisulfate and ammonium bisulfate were the weakest Pas, with 74.54 and 77.13% iron removal efficiency, respectively.

**Figure 4:**
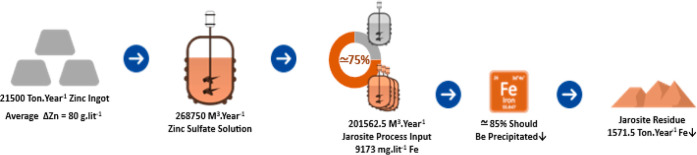
Potential beneficial route for the BZSC plant.

The average BZSC’s production of zinc ingots and ZSS is 21,500 tons and 268,750 m^3^ per year, respectively. 75% of the zinc sulfate is directed to the input of the jarosite precipitation stage, which, considering the beneficial changes by using ammonium sulfate PAs at this stage, would have a significant impact on the plant’s total efficiency (Figure 4). As explained by BZSC’s production instructions, about 80 to 85% of the iron in the solution entering the JPP must be removed from the solution. The rest must remain in the solution and transfer to the neutral leaching stage to remove other impurities in conjunction with iron during the coprecipitation process and prepare a suitable solution for transferring to downstream processes.

Among all of these chemicals, sodium sulfate is considered the cheapest compound; thus, it is the most used PA through JPP in the zinc hydrometallurgy processes. However, in the industrial JPP, based on the PAs’ prices and their performance in removing iron from the solution, there are two very important parameters, including the amount of PA consumption and the amount of sulfuric acid generation during JPP. PA consumption directly impacts economic issues as an important additive in the JPP stage. On the other hand, it can be realized that for each mole of iron removed, 2 mol of hydrogen ions (H^+^) would be generated when sulfates are used (eq 5), and this amount varies from one PA to another. Thus, the amount of sulfuric acid produced in the JPP process varies depending on the type of PA used. The increase in environmental acidity leads to a significant reduction in the JPP rate and iron removal. Therefore, continuous neutralization of the generated sulfuric acid during jarosite formation is imperative to permit effective jarosite precipitation.

The common neutralizing agent in zinc smelters is ZC, where ZnO is the main constituent that consumes sulfuric acid and reduces the acidity of the solution. It should be noted that ZC also contains soluble and insoluble components, which will contaminate the ZSS and jarosite residue, respectively. Particularly, zinc ferrite (ZnO·Fe_2_O_3_), which is present in the ZC, is a problematic compound. The zinc ferrite does not dissolve under the conditions utilized for JPP and leads to zinc losses since the jarosite residue is stockpiled as process waste. Thus, using a PA that produces less sulfuric acid during the jarosite formation reaction can greatly improve the efficiency of the process. Moreover, the ZC used in JPP is not completely dissolved, and part of the zinc metal enters the jarosite residue. Therefore, these phenomena must be considered for choosing the most efficient PA to reduce zinc losses and improve the efficiency of iron rejection through zinc extraction. Table 5 shows the amount of consumption of each PA and ZC used in the experiment to neutralize the generated sulfuric acid and adjust the medium reaction acidity.

**Table 5:** Amount of Consumption of PAs and ZC during Experiments

type	compound	molar mass	precipitating agent usage			zinc calcine usage		
			ion (mol/lit)	compound (mol/lit)	compound (g/lit)	initial (g/lit)	during exp. (g/lit)	total (g/lit)
hydroxide	(NH_4_)OH	35.05	0.03	0.03	1.05^a^	25.80	15.44	41.24
	NaOH	40.00	0.03	0.03	1.20	25.80	15.29	41.09
carbonate	(NH_4_)_2_CO_3_	96.09	0.03	0.015	1.44	25.80	15.33	41.13
	Na_2_CO_3_	105.99	0.03	0.015	1.59	25.80	15.17	40.97
bicarbonate	(NH_4_)HCO_3_	79.06	0.03	0.03	2.37	25.80	15.21	41.01
	NaHCO_3_	84.01	0.03	0.03	2.52	25.80	14.97	40.77
sulfate	(NH_4_)_2_SO_4_	132.14	0.03	0.015	1.98	25.80	19.29	45.09
	Na_2_SO_4_	142.04	0.03	0.015	2.13	25.80	18.93	44.73
bisulfate	(NH_4_)HSO_4_	115.11	0.03	0.03	3.45	25.80	20.93	46.73
	NaHSO_4_	120.06	0.03	0.03	3.60	25.80	20.59	46.39

a In the case of ammonium hydroxide, due to being a solution, the calculated volume was added to the reaction medium according to its purity and specific gravity.

**Figure 5:**
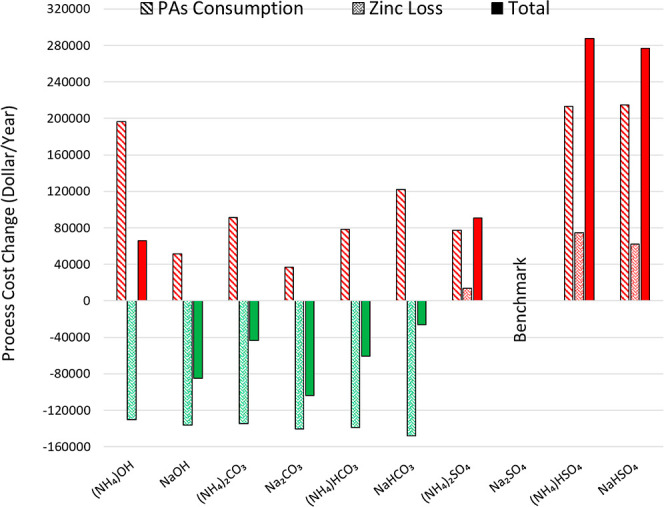
Comparison of the effect of various PAs on the economy of the JPP.

According to the PA and ZC consumption results (Table 5), an economic comparison (Figure 5) of various PAs demonstrated that bisulfates, in addition to their high cost, cause the highest zinc losses due to the large production of sulfuric acid through the JPP (their usage enforces a high cost on the iron removal process). Moreover, bisulfates had the lowest efficiency in removing iron from ZSS among all the examined PAs (Figure 3); thus, using bisulfates has no economic or technical justification.

**Figure 6:**
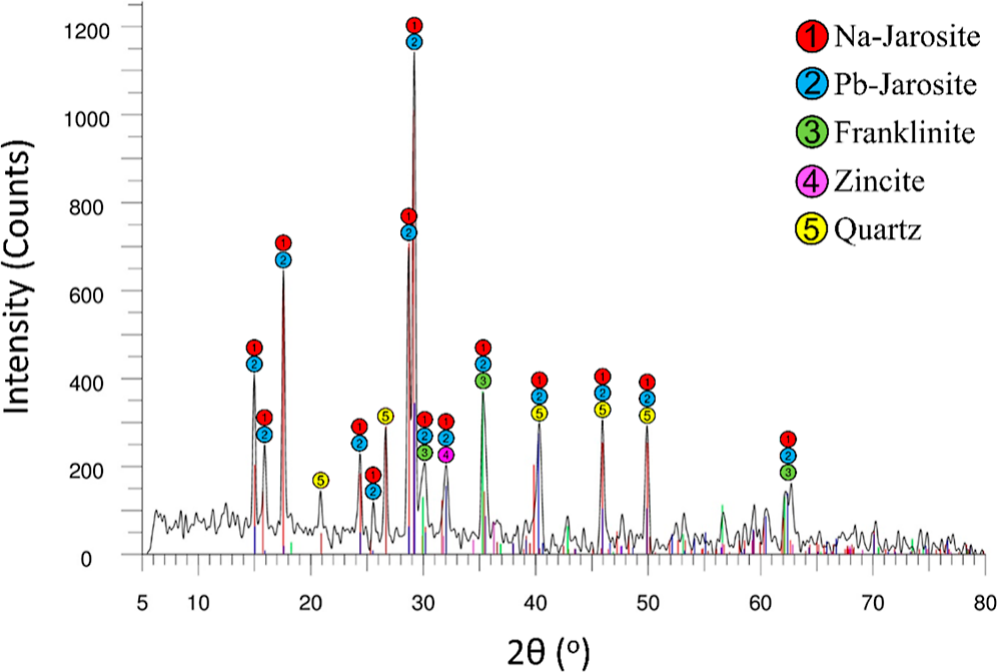
XRD patterns of precipitated jarosite using sodium carbonate as PA.

**Figure 7:**
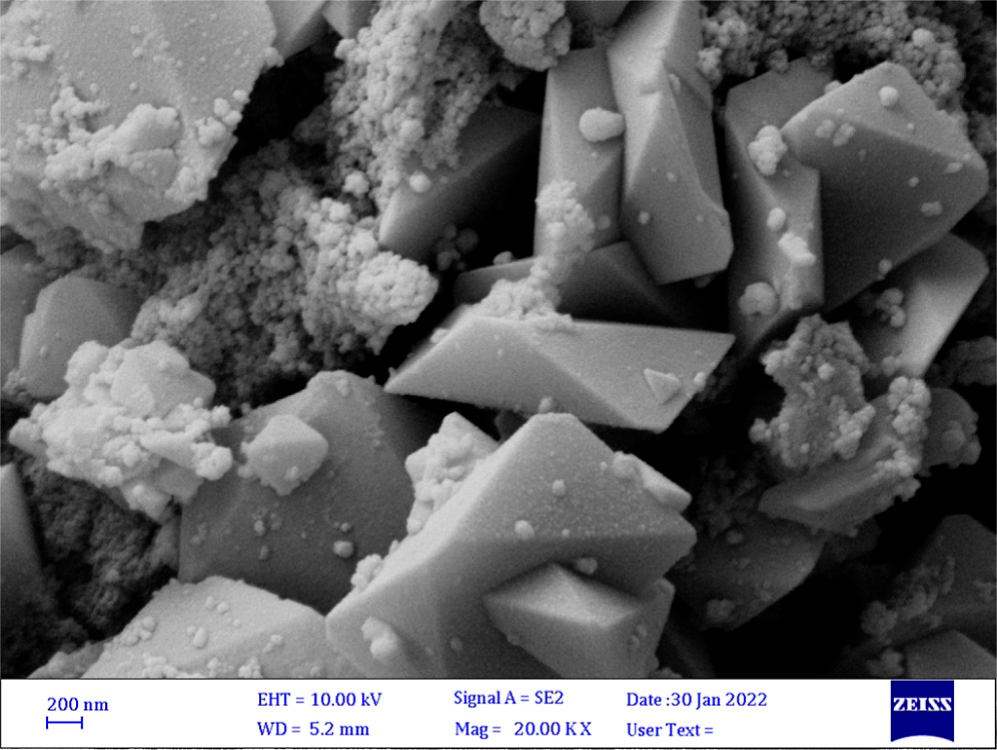
SEM image of precipitated jarosite using sodium carbonate as PA.

**Figure 8:**
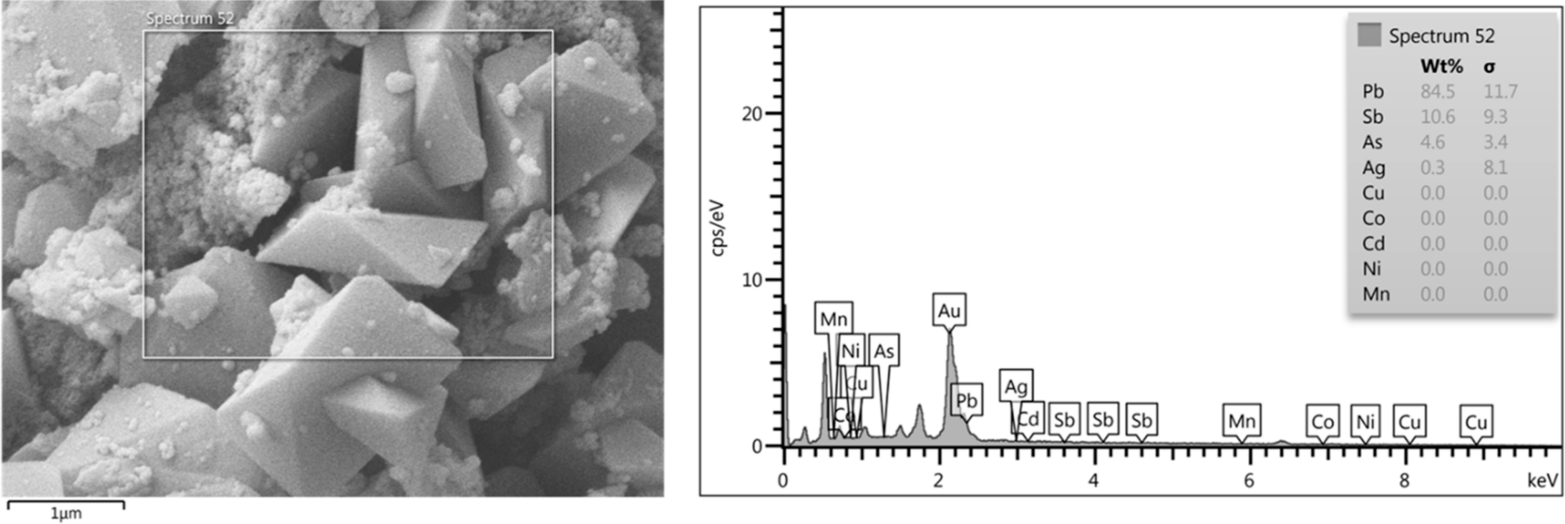
EDS image of precipitated jarosite using sodium carbonate as PA.

Although ammonium hydroxide has the highest performance in removing iron from the solution and reduces the sulfuric acid produced in the process compared to the current situation of the plant, it is not recommended for JPP due to its high cost and severe corrosion. Although other compounds are more expensive than sodium sulfate, they reduce process costs since they produce lower sulfuric acid, reducing ZC consumption and zinc losses. Meanwhile, the use of sodium carbonate, which leads to the formation of sodium jarosite (Figures 6, 7 and 8), has the greatest effect on reducing process costs, which due to its proper performance in iron precipitation from the solution, is preferred to other PAs.

## 4 Conclusions

Exploring various PAs for iron removal in an industrial-scale assessment indicated that regardless of the economic issues, ammonium hydroxide had the highest iron removal efficiency (90.85%). In the same conditions, sodium bisulfate and ammonium bisulfate led to the lowest performances, with 74.54 and 77.13% iron removal efficiency, respectively. Various technical and economic assessments released showed that although sodium sulfate is the cheapest substance among the PAs, it is not the most reasonable PA from an economic point of view for the JPP. Process assessments also highlighted that bisulfates are cost-intensive compounds and have a lower performance efficiency. The industrial-scale process assessment showed that ammonium hydroxide, as a corrosive compound, apart from its high performance in iron removal, is not a suitable alternative to sodium sulfate due to economic and safety issues. Sodium carbonate is more expensive than sodium sulfate; however, it could reduce the amounts of sulfuric acid generation, the consumption of ZC, zinc losses, and finally the process costs. Results demonstrated that sodium carbonate, with an iron removal efficiency of 84.31%, would be a promising alternative to sodium sulfate for jarosite precipitation due to its satisfactory iron removal performance and significant impact on total process cost reduction.
